# Prevalence and Associated Factors of Opioid Withdrawal Syndrome in Adults Admitted to an Intensive Care Unit: A Retrospective Observational Descriptive Study

**DOI:** 10.62641/aep.2387

**Published:** 2026-10-15

**Authors:** Begoña Zalba-Etayo, Clara Serrano-Ferrer, José Luis Cabrerizo-García

**Affiliations:** ^1^Intensive Care Unit, Lozano Blesa University Clinical Hospital, 50009 Zaragoza, Spain; ^2^Faculty of Medicine, University of Zaragoza, 50009 Zaragoza, Spain; ^3^Internal Medicine, Lozano Blesa University Clinical Hospital, 50009 Zaragoza, Spain

**Keywords:** opioid withdrawal syndrome, Clinical Opiate Withdrawal Scale, opioid-related disorders, post-intensive care syndrome

## Abstract

**Background:**

Prolonged opioid administration for pain control and to facilitate tolerance of mechanical ventilation in the intensive care unit (ICU) may lead to tolerance and physical dependence. Following opioid discontinuation, opioid withdrawal syndrome (OWS) may occur. Although this condition has been well described in pediatric populations, its incidence and associated factors in critically ill adults remain uncertain because of diagnostic heterogeneity and the frequent clinical overlap with delirium. The aim of this study was to assess the prevalence of OWS and to identify factors associated with its development in adult patients.

**Methods:**

We conducted a retrospective, descriptive, observational study including patients evaluated at the post-ICU follow-up clinic of Lozano Blesa University Clinical Hospital (Zaragoza, Spain) over a 12-month period. This study investigated the association between multiple opioid medications and adjunctive medications and the occurrence of OWS. Eligible patients had an ICU stay of >7 days, received mechanical ventilation for >48 hours, and had been exposed to opioids. Demographic, clinical, and pharmacological variables were collected, including types of opioids, weight-adjusted cumulative dose, and use of adjuvant medications. OWS was diagnosed during the first 48 hours after opioid discontinuation using a combination of the Clinical Opiate Withdrawal Scale (COWS) and Diagnostic and Statistical Manual of Mental Disorders, Fifth Edition (DSM-5) criteria. Receiver operating characteristic (ROC) curve analyses were performed to evaluate the impact of morphine exposure.

**Results:**

A total of 66 patients were included (mean age 59.9 ± 13.6 years; predominantly male). The leading cause of ICU admission was respiratory disease (48.5%). Mean Acute Physiology and Chronic Health Evaluation II (APACHE II) score at admission was 17.0 ± 6.1, and median ICU length of stay was 19 days. Twenty-two patients (33.3%) developed OWS, which was mild in 90.9% and moderate in 9.1% according to the COWS. No significant differences were observed between groups in baseline characteristics or delirium occurrence. Patients who developed OWS were more likely to have received morphine (100% vs. 75%; *p* = 0.010) and had a higher weight-adjusted cumulative morphine dose (3.15 ± 2.33 vs. 2.21 ± 2.50 mg/kg; *p* = 0.047), whereas no differences were found for fentanyl exposure. In addition, they more frequently required benzodiazepines (68.2% vs. 18.2%), neuroleptics (68.2% vs. 6.8%), and dexmedetomidine (31.8% vs. 4.5%) (*p* < 0.01 for all comparisons). Cumulative morphine dose showed modest discriminatory performance for OWS (area under the curve (AUC) 0.653; *p* = 0.048).

**Conclusions:**

OWS is a common complication among adult ICU survivors, although it is predominantly mild in severity. A higher cumulative dose of morphine was associated with its onset, although no association was found with the total duration of exposure. Clinical overlap with delirium and the reactive use of psychoactive medications may contribute to under-recognition of this condition, underscoring the need for structured OWS screening during ICU stay and in post-intensive care syndrome (PICS) follow-up.

## Introduction

Advances in intensive care over recent decades have improved survival among critically ill patients but have also brought increasing attention to complications arising not only from the underlying disease itself, but also from the treatments administered during the intensive care unit (ICU) stay. Among these, post-intensive care syndrome (PICS) is particularly relevant, encompassing a range of physical, cognitive, and psychological impairments that persist after hospital discharge and adversely affect the quality of life of both patients and their families [1, 2].

Opioid analgesics are commonly used in the ICU and are recommended by most international guidelines as the cornerstone of analgesia in critically ill patients, both for pain control and to facilitate tolerance of mechanical ventilation [3, 4]. However, prolonged administration may lead to tolerance and physical dependence, thereby increasing the risk of iatrogenic opioid withdrawal syndrome (OWS) when treatment is tapered or discontinued. This syndrome is characterized by autonomic, gastrointestinal, neurological, and behavioral manifestations that may interfere with clinical recovery [5].

OWS was first described in the 1990s in pediatric intensive care units, where its incidence and risk factors have been extensively studied [6]. In adults, however, the available evidence remains considerably more limited. Reported incidence rates vary widely, depending on the diagnostic criteria applied and the characteristics of the populations examined [7, 8]. Factors associated with its development include cumulative opioid dose, duration of exposure, and concomitant use of benzodiazepines [7, 8]. Nevertheless, the true magnitude of the problem remains uncertain, mainly for three reasons: the heterogeneity of the diagnostic criteria used; the absence of withdrawal assessment tools specifically validated for critically ill adult patients—although instruments developed for the general population are available, such as the Clinical Opiate Withdrawal Scale (COWS) [9]; and the frequent clinical overlap between withdrawal symptoms and other common ICU-related conditions, such as delirium, anxiety, or agitation [10].

Early recognition of OWS is particularly important in patients at risk of developing PICS, as the persistence of physical and psychological symptoms after ICU discharge may be further aggravated by the consequences of prolonged opioid exposure [1]. However, to date, no studies have evaluated the prevalence of OWS in the specific context of post-ICU follow-up focused on PICS.

Therefore, the aim of the present study was to estimate the prevalence of opioid withdrawal syndrome and to identify the pharmacological factors associated with its development in a cohort of adult ICU survivors.

## Materials and Methods

A retrospective, descriptive, observational study was conducted in the post-ICU follow-up clinic of Lozano Blesa University Clinical Hospital (Zaragoza, Spain). This clinic, dedicated to the early identification and management of sequelae related to ICU admission within the framework of PICS, provides standardized follow-up for patients referred by the intensive care team after hospital discharge.

During the 12-month study period, 66 patients were evaluated in this clinic and screened for inclusion. Eligible patients met the following criteria: ICU length of stay longer than 7 days, invasive mechanical ventilation for more than 48 hours, and exposure to intravenous opioids for at least 72 hours. Among these, 22 patients developed opioid withdrawal syndrome. Exclusion criteria were chronic opioid use prior to ICU admission, pre-existing neurological or psychiatric disease precluding reliable assessment of withdrawal symptoms, and incomplete documentation of sedoanalgesia in the medical record.

The variables collected were grouped as follows:

• Demographic and baseline variables: age, sex, body mass index, and relevant medical history (smoking, alcohol use, and psychiatric history).

• Clinical variables during ICU admission: primary diagnosis, Acute Physiology and Chronic Health Evaluation II (APACHE II) score [11], ICU length of stay, and presence of delirium, diagnosed using either the Confusion Assessment Method for the Intensive Care Unit (CAM-ICU) [12] or the Intensive Care Delirium Screening Checklist (ICDSC) [13].

• Pharmacological variables: type of opioid (morphine, fentanyl, or others), total cumulative dose adjusted for body weight (mg/kg), duration of continuous infusion, and use of adjuvant medications (benzodiazepines, dexmedetomidine, clonidine, antipsychotics, tramadol, and methadone).

OWS was assessed retrospectively by reviewing medical and nursing notes corresponding to the first 48 hours after the initiation of opioid tapering or complete discontinuation. OWS was diagnosed when the patient had a score of ≥5 on the COWS and fulfilled the clinical criteria established by the Diagnostic and Statistical Manual of Mental Disorders, Fifth Edition (DSM-5) [14]. The COWS consists of 11 items that assess common signs and symptoms of withdrawal: resting pulse rate, sweating, restlessness, pupil size, bone or joint aches, runny nose or tearing, gastrointestinal upset, tremor, yawning, anxiety or irritability, and gooseflesh skin. Each item is scored on an ordinal scale (most items range from 0 to 4 or 0 to 5, depending on the item), and the sum of the individual scores provides a total score ranging from 0 to 48.

Severity was classified according to the COWS score as mild (5–12), moderate (13–24), or severe (≥25). It should be noted that opioid discontinuation was performed gradually according to the clinical judgment of the treating team, and no standardized weaning protocol was in place during the study period.

### Statistical Analysis

Continuous variables were expressed as the mean ± standard deviation or median (interquartile range), depending on their distribution, whereas categorical variables were reported as absolute frequencies and percentages. Comparisons between groups (OWS present vs. absent) were performed using Student’s *t*-test or the Mann–Whitney U test for continuous variables, and the chi-square test or Fisher’s exact test for categorical variables.

To evaluate the discriminatory ability of morphine exposure for predicting the occurrence of withdrawal syndrome, a receiver operating characteristic (ROC) curve analysis was performed. The state variable was coded as 1 = presence of withdrawal, 0 = absence. The continuous test variables entered the model were the total hours of morphine administration and the cumulative morphine dose.

The curve was constructed by plotting sensitivity (proportion of true positives) on the Y-axis against the False Positive Rate (1 – Specificity) on the X-axis, evaluating all possible thresholds (cut-off points) of the test variables.

The area under the curve (AUC) and its corresponding 95% confidence interval (CI) were calculated using DeLong’s non-parametric method, and the optimal cut-off point for each variable was estimated based on Youden’s index (Sensitivity + Specificity – 1).

A two-sided *p* value of ≤0.05 was considered statistically significant. Statistical analyses were performed using SPSS version 25.0 (IBM Corp., Armonk, NY, USA).

## Results

During the study period, 66 patients evaluated in the post-ICU follow-up clinic met the selection criteria and were included in the analysis. Mean age was 59.9 ± 13.6 years, and most patients were male. Regarding baseline characteristics, 22.7% were smokers, 9.1% reported alcohol use, and 13.6% had a psychiatric history.

The main reason for ICU admission was respiratory disease, accounting for 48.5% of cases, followed by sepsis (21.2%) and postoperative care (15.2%). Delirium occurred in 53% of patients during their ICU stay. The mean APACHE II score at admission was 17.0 ± 6.1, and the median ICU length of stay was 19 days [P25–P75: 13.75–30.25].

Overall, 22 patients (33.3%) met criteria for OWS, whereas 44 (66.7%) did not develop compatible symptoms. According to the COWS, OWS severity was mild in 90.9% of cases and moderate in 9.1%; no severe cases were identified. Comparative analysis showed no statistically significant differences between patients with and without OWS in terms of age, sex, smoking or alcohol use, psychiatric history, primary diagnosis, APACHE II score, ICU length of stay, or occurrence of delirium during admission (Table 1).

**Table 1.** **Characteristics of the sample**.

Characteristics		OWS present (N = 22)	OWS absent (N = 44)	*p*
Age, years $\bar{x}$ (SD)		57.5 (18.3)	61.1 (11.1)	0.326
Gender, male n (%)		15 (68.2%)	22 (50%)	0.161
Alcohol consumption, n (%)		1 (4.5%)	5 (11.4%)	0.661
Smoking, n (%)		6 (27.3%)	9 (20.5%)	0.781
Psychiatric history, n (%)		4 (18.2%)	5 (11.4%)	0.447
Main diagnosis, n (%)				0.786
	Cardiac pathology	1 (4.5%)	3 (6.8%)	
	Respiratory pathology	13 (59.1%)	19 (43.2%)	
	Sepsis	4 (18.2%)	10 (22.7%)	
	Liver disease	0 (0 %)	1 (2.3%)	
	Polytrauma	2 (9.1%)	3 (6.8%)	
	Postoperative	2 (9.1%)	8 (18.2%)	
Delirium in ICU, n (%)		14 (63.6%)	21 (47.7%)	0.222
APACHE II, $\bar{x}$ (SD)		15.7 (6.4)	17.6 (5.9)	0.236
Mechanical ventilation, $\bar{x}$ (SD)		17.18 (14.2)	20.5 (21.9)	0.728
ICU stay, median (IQR)		17.5 (12–24)	19 (14–31)	0.310

Data are presented as mean ± standard deviation (SD) or n (%). OWS, opioid withdrawal syndrome; ICU, intensive care unit; APACHE II, Acute Physiology and Chronic Health Evaluation II.

Regarding opioid exposure, morphine use was significantly more frequent in the OWS group than in the non-OWS group (100% vs. 75%; *p* = 0.010). In addition, the weight-adjusted cumulative morphine dose was higher among patients who developed OWS (3.15 ± 2.33 vs. 2.21 ± 2.50 mg/kg; *p* = 0.047) (Table 2).

**Table 2.** **Opioid treatment**.

Opioid treatment	OWS present (N = 22)	OWS absent (N = 44)	*p*
Morphine, n (%)	22 (100%)	33 (75%)	0.010
Morphine hours continuous infusion, h (SD)	286.64 (226)	219.38 (269)	0.600
Total morphine dose, mg (SD)	266.62 (188)	189.72 (220)	0.400
Total morphine dose/weight, mg/kg (SD)	3.15 (2.33)	2.21 (2.5)	0.047
Fentanyl, n (%)	10 (45.4%)	23 (52.3%)	0.602
Fentanyl hours continuous infusion, h (SD)	38.14 (109)	89.4 (189)	0.409
Total fentanyl dose, mcg (SD)	1138.73 (5096)	11788.78 (28,464)	0.226
Total fentanyl dose/weight, mcg/kg (SD)	17.73 (79)	122.34 (273)	0.254

OWS, opioid withdrawal syndrome; SD, standard deviation.

By contrast, no statistically significant differences were observed between groups with respect to fentanyl use, duration of administration, total cumulative dose, or weight-adjusted dose (Table 2).

Regarding adjuvant treatment, patients with OWS more frequently received benzodiazepines (68.2% vs. 18.2%; *p* < 0.001), dexmedetomidine (31.8% vs. 4.5%; *p* = 0.004), antipsychotics (68.2% vs. 6.8%; *p* < 0.001), tramadol (13.6% vs. 0%; *p* = 0.014), and clonidine (13.6% vs. 0%; *p* = 0.018). Methadone use was anecdotal and did not differ significantly between groups (Table 3).

**Table 3.** **Adjunctive treatment**.

Medication	OWS present (N = 22)	OWS absent (N = 44)	*p*
Benzodiazepines	15 (68.2%)	8 (18.2%)	<0.001
Dexmedetomidine	7 (31.8%)	2 (4.5%)	0.004
Antipsychotics	15 (68.2%)	3 (6.8%)	<0.001
Tramadol	3 (13.6%)	0 (0%)	0.014
Clonidine	3 (13.6%)	0 (0%)	0.018
Methadone	1 (4.5%)	0 (0%)	0.175

Data are presented as n (%). Percentages refer to the proportion within each group (OWS present or OWS absent). Statistical comparisons were performed using the chi-square test or Fisher’s exact test, as appropriate. OWS, opioid withdrawal syndrome.

ROC curve analysis showed that the weight-adjusted cumulative morphine dose had limited discriminatory ability for the development of opioid withdrawal syndrome (AUC = 0.653; 95% CI: 0.517–0.790; *p* = 0.048). The optimal cut-off point identified by Youden’s index was 2.10 mg/kg (sensitivity 66.7%; specificity 39.5%; Youden’s J = 0.27). However, given the modest AUC, this threshold has limited clinical utility for individual risk stratification at the bedside.

The duration of continuous morphine infusion did not demonstrate statistically significant discriminatory ability (AUC = 0.625; 95% CI: 0.489–0.760; *p* = 0.108) (Fig. 1; Table 4).

**Fig. 1.**
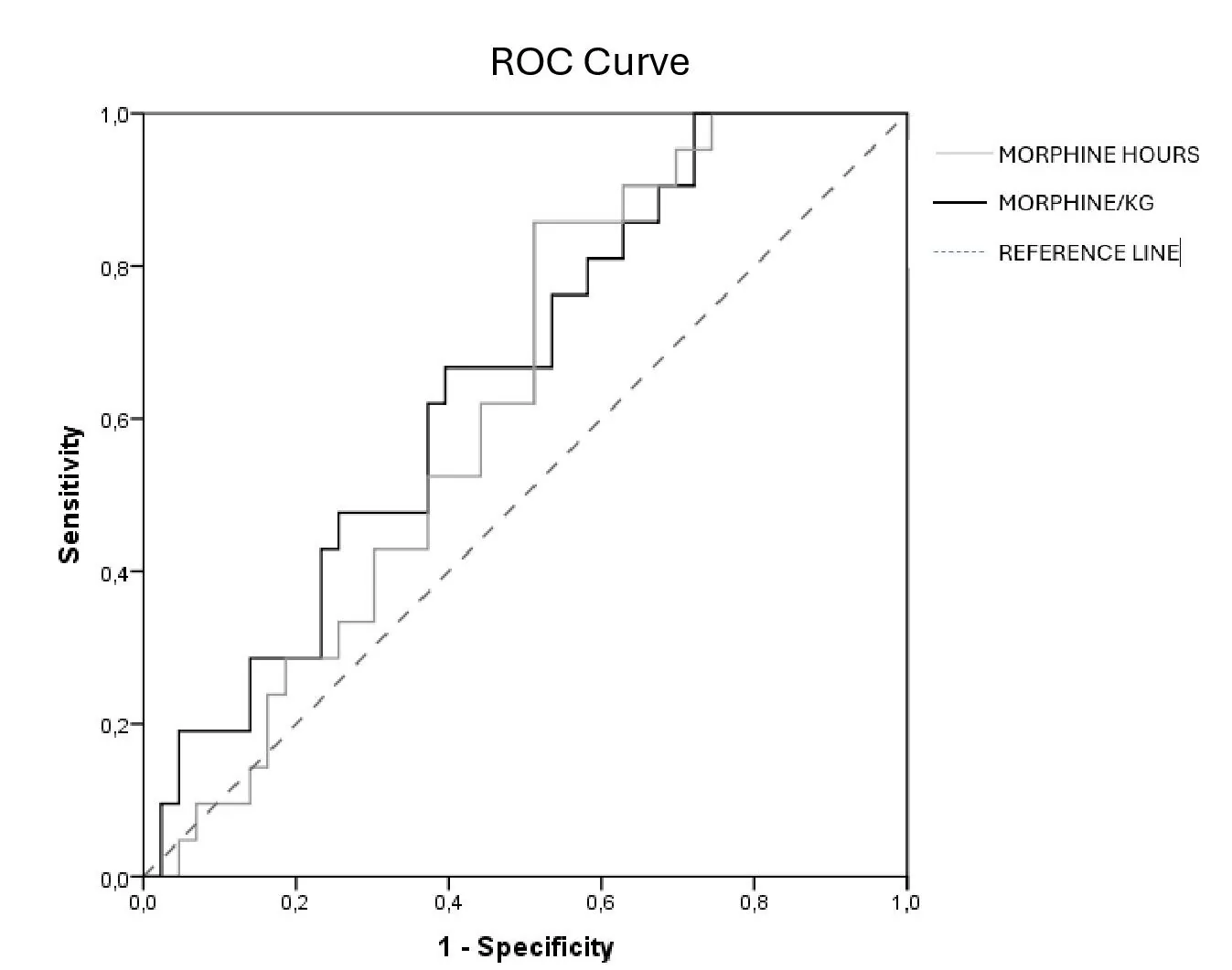
**AUC Morphine/OWS**.

**Table 4.** **Area under the curve**.

Morphine data	Area	Std. Error^a^	Asymptotic Sig.^b^	Asymptotic 95% confidence interval	
				Lower bound	Upper bound
Morphine hours	0.625	0.069	0.108	0.489	0.760
Morphine/kg	0.653	0.069	0.048	0.517	0.790

^a^Standard error; ^b^Asymptotic significance.

## Discussion

The present study examined the occurrence of iatrogenic opioid withdrawal syndrome (OWS) during ICU admission, in a cohort of patients undergoing post-ICU follow-up due to their risk of developing PICS. The main finding was that one-third of the patients included (33.3%) met criteria compatible with OWS, a proportion consistent with previous reports [15], although most cases were classified as mild according to the COWS. This prevalence falls within the range previously described in critically ill adults (16–55%) [5, 7, 8, 16], supporting the view that OWS is not a complication exclusive to pediatric populations, but rather a clinically relevant and probably under-recognized condition in critically ill adults.

In our cohort, patients who developed OWS were more likely to have received morphine and had a significantly higher weight-adjusted cumulative morphine dose. Although the discriminatory performance of this variable was modest (AUC = 0.653), this finding suggests that cumulative morphine burden may play a relevant role in the development of the syndrome, in line with previous studies in critically ill adults [5, 7, 16] and with the available evidence in pediatric populations [8].

By contrast, no significant differences were observed with respect to fentanyl use. This finding should be interpreted cautiously: although pharmacokinetic differences between opioids may exist—such as the absence of clinically relevant active metabolites with fentanyl compared with morphine—it is also possible that this result was influenced by the sample size, the heterogeneity of sedation and analgesia strategies, and the lack of systematic conversion to morphine-equivalent doses. Therefore, these results should be interpreted with caution and do not allow definitive conclusions to be drawn regarding whether fentanyl carries a lower risk of OWS in this population.

Although we found no significant differences between groups in available markers of illness severity (APACHE II score, ICU length of stay, duration of mechanical ventilation, and delirium occurrence), we cannot rule out residual confounding by unmeasured factors such as depth of sedation, individual pain response, opioid metabolism variability, or clinician-specific sedation practices. The lack of systematic recording of sedation depth (e.g., Richmond Agitation-Sedation Scale (RASS) scores) and detailed opioid tapering protocols limits our ability to distinguish whether higher morphine exposure is a direct risk factor for OWS or a marker of greater illness severity requiring deeper sedation.

Therefore, the observed association between cumulative morphine dose and OWS should be interpreted cautiously as a descriptive finding rather than a confirmed independent causal risk factor. Larger prospective studies with systematic collection of sedation depth, pain scores, opioid tapering protocols, and other potential confounders are needed to determine whether morphine exposure per se increases the risk of OWS, or whether it is primarily a marker of greater clinical complexity.

Patients with OWS more frequently received benzodiazepines, antipsychotics, dexmedetomidine, clonidine, and tramadol. However, this association should not be interpreted as evidence of a direct causal relationship. In clinical practice, these drugs are commonly used reactively in response to symptoms such as agitation, autonomic hyperactivity, insomnia, or delirium, all of which are frequent in the setting of OWS. Their greater use therefore probably reflects the greater clinical complexity of these patients rather than an independent etiological role. This issue highlights the limitation imposed by our sample size on the identification of clear independent associations, as there is significant collinearity among pharmacological variables, which, coupled with the limited number of events, precluded the performance of a robust multivariable analysis.

One of the main challenges in identifying OWS in critically ill adults is its extensive clinical overlap with hyperactive delirium and other neuropsychiatric syndromes commonly encountered in the ICU. Manifestations such as agitation, tachycardia, hypertension, diaphoresis, insomnia, or gastrointestinal symptoms may be attributed indistinctly to withdrawal, delirium, inadequately controlled pain, hypoxia, sepsis, or metabolic disturbances [4, 7, 17, 18, 19]. In our cohort, more than half of the patients experienced delirium during ICU admission, illustrating the complexity of the differential diagnosis. This challenge is further compounded by the lack of tools specifically validated for diagnosing OWS in critically ill adults: although the COWS is widely used in other settings, its application in the ICU has limitations, particularly in patients with impaired consciousness, mechanical ventilation, or concomitant sedation [13, 14].

The growing interest in PICS has highlighted the importance of identifying modifiable factors during and after ICU admission that may influence long-term recovery [1, 15, 20, 21]. Survivors of critical illness frequently experience physical, cognitive, and psychological impairment, including anxiety, depression, and post-traumatic stress symptoms [20, 21, 22, 23]. Although our study did not longitudinally assess the impact of OWS on the subsequent development of PICS, it is plausible that unrecognized or late-treated withdrawal may contribute to a greater neuropsychiatric symptom burden during recovery.

In this context, our findings support the incorporation of structured OWS assessment in critically ill patients exposed to prolonged opioid therapy. Such an approach should combine specific withdrawal scales (COWS), assessment of sedation level using the RASS, and systematic delirium screening with CAM-ICU or ICDSC [24], together with an individualized evaluation of the pharmacological context, including opioid type, cumulative dose, duration of exposure, tapering rate, and use of adjuvant medications. Experience in pediatric populations with nurse-driven sedoanalgesia protocols has shown promising results in reducing cumulative opioid exposure and preventing OWS [25], suggesting that similar strategies could be explored in critically ill adults.

The strengths of this study include its novel focus on OWS in the specific setting of post-ICU follow-up aimed at PICS, an area that has so far received little attention; the use of a dual diagnostic approach (COWS + DSM-5), which provides greater methodological rigor; and the inclusion of detailed clinical and pharmacological variables.

### Limitations

Selection bias constitutes a significant limitation of this study. Our cohort consists exclusively of ICU survivors who were referred to and attended our post-ICU follow-up clinic. This design systematically excludes patients who died in the ICU, those who were not referred for follow-up, and those unable or unwilling to attend. Consequently, the reported prevalence should be interpreted as specific to this selected population of long-stay ICU survivors, rather than as a population-wide estimate. Furthermore, the clinical heterogeneity of our cohort, which included diverse admission diagnoses, may have influenced the results and limited our ability to conduct subgroup analyses.

The retrospective, single-center design limits the generalizability of our findings and may have introduced information bias.

Finally, the small sample size restricted our statistical power and precluded the performance of a robust multivariable analysis. Therefore, our findings should be interpreted primarily as descriptive and hypothesis-generating, and they require validation in larger, prospective multicenter studies.

## Conclusions

OWS is a prevalent, mostly mild, and likely underdiagnosed complication in adult ICU survivors due to its overlap with delirium. Although cumulative morphine dose is associated with OWS, its limited predictive performance suggests a multifactorial etiology. A structured diagnostic approach should integrate assessment of opioid exposure (type, dose and tapering) with standardized monitoring of sedation (RASS), delirium (CAM-ICU/ICDSC), and withdrawal (COWS). Such a comprehensive approach is essential to improve early recognition and optimize the management of OWS within the broader PICS framework.

## Availability of Data and Materials

Available from the corresponding author upon request.
